# Techno-economic evaluation of stillage treatment with anaerobic digestion in a softwood-to-ethanol process

**DOI:** 10.1186/1754-6834-3-21

**Published:** 2010-09-15

**Authors:** Zsolt Barta, Kati Reczey, Guido Zacchi

**Affiliations:** 1 Department of Applied Biotechnology and Food Science, Budapest University of Technology and Economics, Szt Gellért tér 4, H 1111 Budapest, Hungary; 2 Department of Chemical Engineering, Lund University, PO Box 124, S 221 00 Lund, Sweden

## Abstract

**Background:**

Replacing the energy-intensive evaporation of stillage by anaerobic digestion is one way of decreasing the energy demand of the lignocellulosic biomass to the ethanol process. The biogas can be upgraded and sold as transportation fuel, injected directly into the gas grid or be incinerated on-site for combined heat and power generation. A techno-economic evaluation of the spruce-to-ethanol process, based on SO_2_-catalysed steam pretreatment followed by simultaneous saccharification and fermentation, has been performed using the commercial flow-sheeting program Aspen Plus™. Various process configurations of anaerobic digestion of the stillage, with different combinations of co-products, have been evaluated in terms of energy efficiency and ethanol production cost versus the reference case of evaporation.

**Results:**

Anaerobic digestion of the stillage showed a significantly higher overall energy efficiency (87-92%), based on the lower heating values, than the reference case (81%). Although the amount of ethanol produced was the same in all scenarios, the production cost varied between 4.00 and 5.27 Swedish kronor per litre (0.38-0.50 euro/L), including the reference case.

**Conclusions:**

Higher energy efficiency options did not necessarily result in lower ethanol production costs. Anaerobic digestion of the stillage with biogas upgrading was demonstrated to be a favourable option for both energy efficiency and ethanol production cost. The difference in the production cost of ethanol between using the whole stillage or only the liquid fraction in anaerobic digestion was negligible for the combination of co-products including upgraded biogas, electricity and district heat.

## Background

Ethanol produced from sugar, starch and lignocellulosic biomass is a liquid biofuel with the potential to replace some of the liquid fossil fuels used in transportation today. Currently, bio-ethanol is produced from sugar- and starch-containing materials [1]. However, it is clear that the large-scale use of ethanol as fuel will require lignocellulosic biomass to be used as raw material [2]. The conversion of lignocellulosic material to ethanol is more complex than ethanol production from sugar or starch. Although pilot-scale and pre-commercial demonstration plants have been brought into operation recently [3-5], the process concept has not yet been demonstrated on an industrial scale.

Many process alternatives have been proposed for the production of ethanol from lignocellulosic materials. The main difference between them is the way in which cellulose and hemicellulose are hydrolysed to fermentable sugars [6-8]. A process based on enzymatic hydrolysis and fermentation is considered to be a promising alternative for the conversion of lignocellulosic carbohydrates to ethanol [6,9]. Compared with separate enzymatic hydrolysis and fermentation, simultaneous saccharification and fermentation (SSF) has been shown to be less capital intensive and to result in higher overall ethanol yields [10-12]. In order to obtain a high conversion of cellulose in enzymatic hydrolysis the raw material must be pretreated [13]. One of the most thoroughly investigated methods is steam pretreatment, with or without a catalyst [14-17].

The economics of the lignocellulosic ethanol process is highly dependent on the income from co-products [18,19]. During the downstream process the lignin-rich solid residue can be separated, dried and pelletized. Pellets are sold as solid fuel on the residential pellet market [20]. Alternatively, steam and electricity can be generated by burning the solid residue together with the concentrated liquid fraction of the stillage. When the wastewater streams are treated with anaerobic digestion (AD) and aerobic treatment steps, the biogas produced and the sludge formed can also be incinerated on-site in a combined heat and power (CHP) facility [21,22]. Above a certain level of biogas production, upgrading of the biogas can also be an option and the upgraded gas can then be sold as a transportation fuel or injected directly to the gas grid [23,24]. As the overall energy demand (for both heat and electricity) of the lignocellulosic ethanol process decreases the energy output in the form of co-products [20], it must be reduced as much as possible and a high degree of heat integration is therefore required. Replacing energy-intensive process steps by less energy-demanding ones, such as replacing evaporation of the stillage stream by AD, can further decrease the heat demand of the process [21].

In this study, the techno-economic aspects of a spruce-to-ethanol process have been investigated using a process concept based on SO_2_-catalysed steam pretreatment followed by SSF. In Sweden, spruce is considered to be the main alternative as raw material in the conversion of lignocellulosic biomass to ethanol, due to its abundance and the relatively high content of carbohydrates [25,26]. This evaluation was focused on investigating alternative options for stillage treatment, such as AD of the liquid fraction of the stillage or AD of the whole stillage stream. Furthermore, the ways in which various process configurations, with different combinations of co-products, affect the overall process in terms of energy efficiency and production cost has also been studied. Sensitivity analyses were performed with regard to the variations in the price of electricity, upgraded biogas, pellets and district heat in order to obtain a wider view of the relation between the scenarios from an economic perspective. The primary aim was not to determine an absolute ethanol production cost but to develop a useful modelling tool for comparing different process scenarios, for identifying possible process bottlenecks and for the identification of future research activities that have the greatest potential to lower the cost of bio-ethanol production. Comparisons of the cost obtained in this evaluation with those reported in similar studies applying other assumptions should be performed with great care. Differences in technological (for example, capacity, recoveries, yields) and/or in financial parameters (for example, interest rate, depreciation period) can render such comparisons invalid.

## Methods

### Process description - the reference case

**Figure 1:**
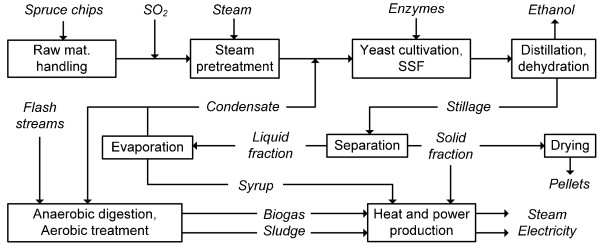
**Overall process scheme for the proposed ethanol plant in the reference case**. Part of the evaporation condensate, together with the condensed flash streams originating from pretreatment and drying, is anaerobically digested followed by an aerobic treatment step. Mat: material; SSF: simultaneous saccharification and fermentation.

The process scheme for the reference scenario is shown in Figure 1. Each step has been previously described in detail [18] and will only briefly be discussed here, focusing mainly on minor modifications. The proposed ethanol plant is assumed to be located in Sweden and to convert 200 000 dry tonnes of spruce chips into 49 416 m^3 ^of ethanol annually. It is run by 28 employees and is assumed to be in operation for 8000 h/year. Live steam is assumed to be available at 20 and 4 bar and secondary steam is used to replace live steam whenever possible.

The dry matter (DM) of spruce contains 43.5% glucan, 12.8% mannan, 2.1% galactan, 5.1% xylan, 1.5% arabinan and 29.4% lignin, all determined by compositional analysis performed in the EU project NILE (contract No. 019882), according to the standardized method of the National Renewable Energy Laboratory (NREL, CO, USA) [27]. The remainder is made up of acetyl groups, extractives and other compounds, which were estimated using the data of a previous study [19]. The DM content is assumed to be 50%. Theoretically, 420 L of ethanol can be produced from the hexose sugars available in a tonne of dry raw material.

The conversion of carbohydrates is carried out in steam pretreatment and in SSF. The conditions used for steam pretreatment (210°C, 2.5% SO_2_, conversion factors for some reactions: glucan to glucose 0.161, glucan to hydroxymethylfurfural 0.013, xylan to xylose 0.674, xylan to furfural 0.220, water-insoluble lignin to water-soluble lignin 0.082) and SSF (conversion factors for some reactions: glucan to glucose 0.756, xylan to xylose 0, glucose to ethanol 0.9, glucose to glycerol 0.005) were based on results recently obtained from experimental work performed at the Department of Chemical Engineering, Lund University, Sweden (unpublished). Part of the evaporation condensate and ammonia are added before pressing the pretreated slurry, in order to adjust the dry matter in the SSF step to 10% water-insoluble solids (WIS) and to neutralize the slurry, respectively. Simultaneous saccharification and fermentation is performed at 37°C with ordinary baker's yeast at a concentration of 3 g DM/L and an enzyme dosage of 10 FPU (filter paper unit)/g WIS. It takes place in 12 agitated non-sterile fermentors each with a volume of 920 m^3^. Yeast is cultivated on the pressed liquid fraction of the diluted pretreated slurry, supplemented with molasses, while the enzymes are purchased.

The ethanol concentration obtained after SSF is 3.5 wt-%. Distillation and molecular sieve adsorption are used to produce pure (99.8 wt-%) ethanol. The distillation step consists of two stripper columns and a rectifier, which are heat integrated by operating at different pressures. Ethanol recovery is assumed to be 99.5% in each column.

The stillage of the stripper columns is separated in a filter press resulting in a solid fraction with a WIS content of 40%. In any scenario where pellets are produced, washing is included in the stillage separation step in order to decrease the sulphur content in the solid fraction. It is assumed that washing - with a soluble solid recovery of 90% in the liquid stream - is sufficient to meet the requirement regarding the sulphur content of pellets.

The liquid fraction of the stillage is concentrated to 60% DM in an evaporation system containing five effects in a forward-feed arrangement. Boiling point elevation was taken into account [28] and overall heat transfer coefficients were estimated to vary between 400 and 2000 W/m^2^°C. Based on the work of Olsson *et al*. [29], it is assumed that, by applying a stripper column after evaporation to remove volatile compounds, part of the evaporation condensate could be recycled to dilute the whole slurry without affecting SSF. The rest of the condensate, together with the condensed flash streams originating from pretreatment and drying, is treated by AD followed by an aerobic treatment step. These steps are described below.

Steam and electricity are generated by burning the concentrated liquid fraction, part of the solid fraction of the stillage, the biogas and the sludge. The generated steam is allowed to expand to 4 bar through a high-pressure turbine system. However, part of the steam is withdrawn at 20 bar for pretreatment and drying. District heating was not included in the reference case. The excess solid residue (87% of the total) - the solid fraction not required for steam generation, is dried in a superheated steam dryer to 88% DM and then pelletized. The secondary steam generated by drying is utilized in the process.

### Alternative stillage treatment scenarios

**Figure 2:**
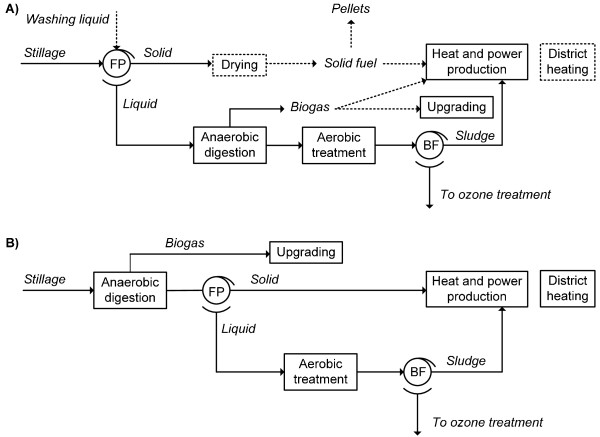
**Alternative stillage treatment scenarios**. Either the liquid fraction of the stillage is anaerobically digested (A), or the whole stillage is fed directly to anaerobic digestion (B). The dotted parts are optional, see Table 1 for details. The wastewater streams, such as the condensed flash streams from pretreatment and drying, are also sent to anaerobic digestion but they are not shown here. BF: belt filter; FP: filter press.

In the case of alternative stillage treatment the heat-demanding evaporation plant was excluded from the ethanol process. These scenarios are summarized in Figure 2 and Table 1. In scenarios A1 to A4 the stillage is separated in a filter press (described above) and the liquid stream is treated by AD in order to produce biogas. The effluent from AD is subjected to an aerobic treatment step. In scenario B the whole stillage is treated directly by AD. However, the effluent of AD is separated and only the liquid fraction is fed to the aerobic treatment step. The same type of filter press as that used for stillage separation is used and it is assumed that the WIS content of the solid fraction is 35%. This solid fraction, comprising lignocellulosic residue and anaerobic sludge, is assumed to be burnt in the CHP plant. In all alternative treatment scenarios sludge separation after the aerobic treatment is performed with a belt filter, which increases the sludge DM to 30%. The pressed sludge is incinerated on-site.

**Table 1:** Differences in stillage processing in the scenarios investigated

Scenario	Washing and drying	Pellet production	Biogas upgrading	Turbine system	Burnt in CHP (besides sludge)	Co-products*	DH
Reference	Yes	Part of sfrac of stillage (dried)	No	HP	Part of sfrac of stillage, syrup, bg	Pellets	No
A1	Yes	Part of SF	Yes	HP	Part of SF, tail gas	Up bg, pellets	No
A2	Yes	TA of SF	No	HP-LP	Bg	Pellets	Yes
A3	No	-	Yes	HP-LP	Sfrac of stillage, tail gas	Up bg	Yes
A4	No	-	No	HP-LP	Sfrac of stillage, bg	-	Yes
B	-	-	Yes	HP-LP	Sfrac of eff, tail gas	Up bg	Yes

*The electricity generated is not included in co-products. Although it is produced in all scenarios, in some cases electricity consumption exceeds production.

Bg: biogas; CHP: combined heat and power; DH: district heating; eff: effluent of anaerobic digestion; HP: high-pressure; LP: low-pressure; SF: solid fuel; Sfrac: solid fraction; TA: total amount; Up: upgraded.

Anaerobic digestion is performed under mesophilic conditions and, hence, the inlet flow is cooled down to 37°C before being fed to the first digester. The assumed degradation factors during AD are: (i) 90% for soluble sugars, organic acids, ethanol, glycerol, enzyme and yeast; (ii) 50% for polysaccharides, extractives, degradation products and water-soluble lignin; and (iii) 0% for water-insoluble lignin. The methane and AD sludge yields are assumed to be 0.35 Nm^3^/kg COD (chemical oxygen demand) removed and 0.03 kg DM/kg COD fed, respectively. In order to obtain the appropriate levels of N and P, solutions of NH_3 _(25%) and H_3_PO_4 _(50%) are added in dosages of 18 g/kg and 4 g/kg COD, respectively. An organic loading rate of 10 kg COD/m^3^/day is applied and, hence, the hydraulic retention time varies between 6.2 and 9.9 days. The specific power required for stirring depends on the scenario and is assumed to be 20 W/m^3 ^for scenarios A1 to A4 and 40 W/m^3 ^for scenario B. The digesters, arranged in series, are continuous stirred tank reactors with a volume of around 3500 m^3 ^each and their number varies between 2 and 12, including the reference scenario. As the water-insoluble lignin was considered to be inert in terms of biogas production, its COD was not included in the COD used to calculate the reactor volume and the demand of chemicals. The biogas is assumed to consist of 50 wt-% methane, 46% CO_2 _and 4% water. The design data and cost for AD were obtained from a supplier of wastewater treatment plants with experience in the treatment of wastewater from the pulp industry (PURAC AB, Lund, Sweden), based on calculated flows and estimated COD content. Biogas upgrading is performed in some scenarios where the biogas is sold as a co-product supplied to the gas grid. In these scenarios all the biogas produced is upgraded using pressure swing adsorption technology. However, 5% of the methane is assumed to be retained in the tail gas that is burnt on-site.

The whole effluent from AD (scenarios A1 to A4), or the separated liquid stream from the AD effluent (scenario B), is first treated aerobically. The organic matter is removed almost entirely and sludge is produced at a yield of 0.3 kg sludge DM/kg organic matter. The last step is ozone treatment, which degrades some phenolic compounds that have not been broken down in the aerobic treatment. The effluent from the ozone treatment is regarded as clean water and is recycled to the process partially or entirely depending on the scenario. The recycled water stream is sterile-filtered before being used to dilute the pretreated slurry. However, the cost of this filter was not included in the economic evaluation.

Similarly to the reference case, in scenario A1 the CHP plant only covers the heat demand of the process, while in scenarios A2 to A4 and B excess heat is produced that is used to generate electricity through an additional low-pressure turbine with a discharge pressure of 0.75 bar. The condensation heat of the outlet steam from the low-pressure turbine and the heat obtained in flue gas condensation are used for district heating. Detailed descriptions of the turbine system and flue gas condensation can be found elsewhere [20]. The return water of 6 bar from the district heating system is heated up from 45°C to 90°C by passing through the flue gas condenser and the turbine condenser.

### Analysis

Mass and energy balances were solved using the commercial flow sheeting program Aspen Plus™, version 2006.5 (Aspen Technology, Inc, MA, USA). Data on the physical properties of biomass components such as polysaccharides and lignin were taken from the NREL database [30]. Aspen HX-Net, version 2006.5 (Aspen Technology) was used to design a near-optimal heat exchanger network, which was implemented in the process model in Aspen Plus. The energy efficiency, based on the lower heating values, is defined as the energy output in the products (ethanol, pellets, biogas, electricity and district heat) divided by the energy input comprising raw material, molasses, enzymes and the fuel equivalent of the electric power requirement, which was calculated using an electricity-to-fuel ratio of 0.4.

The fixed capital investment cost was estimated either with Aspen Icarus Process Evaluator, version 2006.5 (Aspen Technology) setting 2009 as costing year or from vendor quotations. The construction material for all process vessels is assumed to be 304 stainless steel. Working capital was calculated using the recommendation of Peters and Timmerhaus [31] with a slight modification. The annualized fixed capital cost was determined by multiplying the fixed capital investment by an annualization factor of 0.110, corresponding to a 15-year depreciation period and an interest rate of 7% [32]. The annualized working capital is the product of working capital investment and interest rate.

All costs are presented in Swedish kronor (SEK, 1 euro ≈ 10.5 SEK; US$ 1 ≈ 7.3 SEK). The purchase price of enzymes is assumed to be 28.5 SEK/million FPU, which was obtained by multiplying an old estimate of cellulase price [33] by 1.5. The costs of raw material, chemicals, utilities, labour, insurance and maintenance, and the revenues from co-products have been reported in recent studies [19,20]. The total cost of aerobic and ozone treatment, the cost of biogas upgrading and the selling price of upgraded biogas are assumed to be 0.5 SEK/kg COD, 100 and 600 SEK/MWh upgraded biogas, respectively. Other costs comprise labour, insurance and maintenance.

## Results and discussion

### Process design and energy efficiency

Important process details of the scenarios are given in Table 2. The largest biogas production was obtained in scenario B, due to the increased flow of polysaccharides, soluble sugars and other degradable components. In the reference case the organic matter fed to AD consisted of only volatile substances originating from the pretreatment, evaporation and drying steps. The overall degradation factor - the COD removed divided by the COD fed - including or excluding the water-insoluble lignin, varied between 28% and 60% or between 58% and 66%, respectively. The slight difference in the biogas production in scenarios A1 to A4 was due to the drying step, since the condensed outlet steam from the dryer increased the COD flow of others for scenarios A1 and A2.

**Table 2:** Process details of the various scenarios

		Reference	A1	A2	A3	A4	B
Organic matter to anaerobic digestion	t COD/h	1.9	9.7	9.7	9.5	9.5	30.9
Polysaccharides	t COD/h	-	0.1	0.1	0.1	0.1	2.4
Soluble sugars	t COD/h	-	2.2	2.2	2.2	2.2	2.4
WIL	t COD/h	-	0.9	0.9	0.9	0.9	17.8
Others*	t COD/h	1.9	6.5	6.5	6.3	6.3	8.2
Organic matter removed	t COD/h	1.1	5.8	5.8	5.7	5.7	8.5
COD removed/COD fed incl. WIL	%	58	60	60	60	60	28
COD removed/COD fed excl. WIL	%	58	66	66	66	66	65
Energy flow† of raw biogas	MW	3.8	19.9	19.9	19.5	19.5	29.4
Energy flow† of upgraded biogas	MW	-	18.9	-	18.5	-	27.9
							
Overall heat duty of the process	MW	27.8	17.6	17.6	17.9	17.9	17.1
Total power demand of the process	MW	4.1	4.4	4.5	3.8	4.0	4.5
Electricity produced in CHP	MW	5.9	3.0	4.0	11.7	17.1	9.8
District heat	MW	-	-	4.7	34.1	48.9	31.1
							
Energy flow† of pellets‡	MW	48.5	39.3	56.2	-	-	-

The following input energy flows (in MW, based on lower heating values) were the same in all the scenarios: raw material 105.1; molasses 1.7; and enzymes 0.7. The positive and negative differences between power demand and electricity produced indicate purchasing or selling of electricity, respectively. A summary of the scenarios is given in Table 1.

* Includes organic acids, ethanol, glycerol, enzyme, yeast, extractives, degradation products and water-soluble lignin, depending on the scenario.

^† ^Based on lower heating values.

^‡^Only the fraction of the solid fuel sold is given here, i.e. the fraction incinerated on-site is excluded.

CHP: combined heat and power production; COD: chemical oxygen demand; WIL: water-insoluble lignin.

Removing the evaporation step decreased the heat duty of the process considerably, from 27.8 MW to 17.1-17.9 MW. The ethanol production was the same in all scenarios (6177 L/h); hence the specific heat demands were reduced from 16.2 to 9.9-10.4 MJ/L ethanol. The minor increase in the overall heat duty obtained by removing the drying step from the process (scenarios A3 and A4 compared with scenarios A1 and A2) is not inconsistent. On the one hand, it is due to the different heat-exchanger networks with or without drying. On the other hand, the superheated steam drying produces almost as much heat as it requires (data not shown) and the secondary steam leaving the drying step can be utilized at other stages in the process.

The electricity produced on-site varied considerably, unlike the total power requirement of the process. The highest electricity production was obtained for scenario A4, where both the biogas and the solid residue are burnt in the CHP plant. In scenarios A1 and A2 the power produced was lower than the power demand in the process, thus requiring the purchase of electricity, while in all other scenarios a surplus of electricity was produced that could be sold. The largest pellet production was obtained for scenario A2, where, unlike other scenarios, the entire solid residue was pelletized. In the case of district heating, the more heat that can be delivered, the more difficult it is to find an appropriate plant location. There are currently less than 10 district heating systems in Sweden [20] that match the district heat duty for scenario A4 (48.9 MW). Scenarios with district heat production of around 30 MW may be easier to implement as there are more systems operating on this scale.

**Figure 3:**
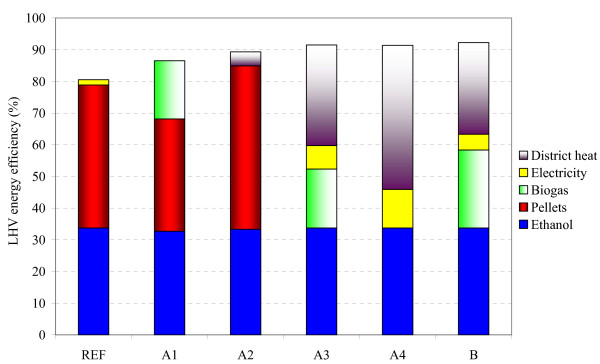
**Overall energy efficiency, based on lower heating values (LHV), expressed as percentage of the input**. A summary of the scenarios is given in Table 1. REF: reference case.

The reference case had significantly lower overall energy efficiency (81%) than the alternative stillage treatment scenarios (87-92%), see Figure 3. This was due to the difference in the overall heat demand, since the reduction in the heat demand of the ethanol process increased the energy output in the co-products. The energy efficiency obtained for ethanol varied between 33% and 34%, which emphasizes the importance of co-products in an energy-efficient process. If the upgraded biogas was used as a fuel as well as the ethanol, 51%-58% of the energy input would be recovered as transportation fuel.

All the scenarios including district heating (A2 to A4, B) had a higher overall efficiency than scenarios without district heating (reference case and A1). Concordant results were obtained in a similar previous study, in which various process configurations for ethanol production from spruce, based on evaporation of the liquid fraction of stillage, were investigated [20]. The overall energy efficiency of the scenarios including district heating varied only slightly (89%-92%), whereas the energy efficiency would differ significantly (46%-85%) if the district heating was excluded from these scenarios (for example, during the summer, when the released heat has to be removed by cooling water).

### Capital investment and ethanol production costs

The lowest and highest total capital investment costs were obtained for scenarios A1 (1199 million SEK) and A4 (1360 million SEK), respectively (Table 3). The main contributors to the total direct cost were the pretreatment unit, the fermentation stage (yeast cultivation and SSF) and the CHP plant. The highest cost for CHP was obtained in scenario A4, where it constituted 44% of the total direct cost. Anaerobic digestion had the highest direct cost in scenario B, where the whole stillage stream was anaerobically digested. When the COD flow of lignin was included in the COD flow used to design AD, the direct cost of AD increased to 225 million SEK in scenario B. The ratio of working capital to fixed capital varied between 2.4% and 6.4% in the scenarios investigated.

**Table 3:** Breakdown of the total capital investment cost in million Swedish Kronor (SEK)

	Reference	A1	A2	A3	A4	B
Raw material handling	10	10	10	10	10	10
Pretreatment	115	115	115	115	115	115
Yeast cultivation and SSF	119	119	119	119	119	119
Distillation	47	47	47	47	47	47
Separation^1^	26	26	26	26	26	25
Evaporation	47	-	-	-	-	-
Drying and pellet production	42	45	47	-	-	-
CHP^2^	155	110	136	266	343	248
Storage	33	28	28	28	28	29
Heat exchanger network	18	11	11	11	11	12
AD	15	75	75	74	74	111
Total direct cost	628	586	613	696	773	716
Total indirect cost	578	551	557	539	556	557
Fixed capital investment	1206	1137	1170	1236	1329	1273
Working capital	69	62	75	32	32	32
Total capital investment	1275	1199	1246	1268	1360	1305

^1 ^Refers to stillage separation in the reference case and in scenarios A1-A4 and to separation of the effluent of AD in scenario B.

^2 ^Includes the flue gas condenser for scenarios with district heating (A2-A4 and B).

A summary of the scenarios is given in Table 1.

AD: anaerobic digestion; CHP: combined heat and power production; SSF: simultaneous saccharification and fermentation.

**Figure 4:**
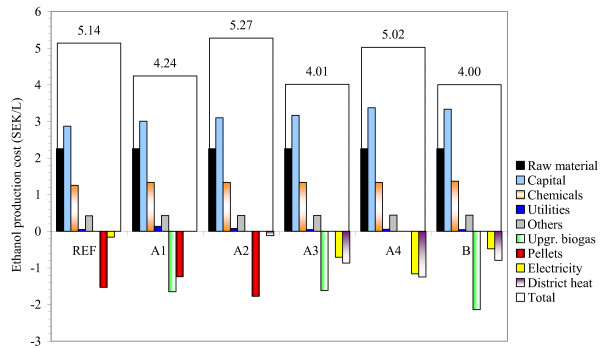
**Breakdown of ethanol production cost in SEK/L**. Chemicals include enzymes. 'Others' refers to the cost of labour, insurance and maintenance. The cost of aerobic and ozone treatment was added to the capital cost: the cost of upgrading was taken into account by reducing the income of upgraded biogas. A summary of the scenarios is given in Table 1. REF: reference case; SEK: Swedish kronor; Upgr: upgraded.

The major contributors to ethanol production cost were the cost of capital, raw material and chemicals, in descending order (Figure 4), whereas the utilities and other costs made minor contributions. The alternative stillage treatment increased the cost of capital, chemicals and other costs compared with the reference case. The highest cost of utilities was obtained for scenarios A1 and A2, where electricity had to be purchased. The favourable combinations for the co-products of the alternative stillage treatment scenarios were: (i) upgraded biogas and pellets (scenario A1); and (ii) upgraded biogas, electricity and district heat (scenarios A3 and B). However, the combination of (a) pellets and district heat (scenario A2) and (b) electricity and district heat (scenario A4) resulted in lower co-product income.

With respect to the production cost of ethanol, the alternative stillage treatment configurations proved to be good options in some cases. Scenarios A1, A3 and B resulted in low ethanol production costs (4.00-4.24 SEK/L), while scenarios A2 and A4 were less economical (5.27 and 5.02 SEK/L, respectively). Scenario A2 had an even higher ethanol production cost than the reference case (5.14 SEK/L). Scenarios A1 and A2 had almost the same costs. However, they differed greatly in their co-product incomes. Producing 4.7 MW district heat at a price of 280 SEK/MWh and 56.2 MW pellets at 195 SEK/MWh (scenario A2) resulted in much less income than producing 18.9 MW upgraded biogas with a selling price of 600 SEK/MWh and an upgrading cost of 100 SEK/MWh together with 39.3 MW pellets (scenario A1; Table 2).

The performance of the AD step is very uncertain due to a lack of experimental data on the continuous treatment of stillage from a wood-based ethanol process. Therefore two sub-scenarios were developed from scenario B and the ethanol production cost was determined. In one sub-scenario the COD flow of water-insoluble lignin was included in the COD flow used to design the anaerobic digesters and for calculating the demand of chemicals in AD (however, the conversion of water-insoluble lignin was maintained at 0%) and the ethanol production cost increased to 4.71 SEK/L, compared with 4.00 SEK/L in scenario B. In the other sub-scenario it was assumed that the polysaccharides in the solid fraction were not converted to biogas - their degradation factors were set to 0%. Since the COD flow into AD was the same as that in scenario B, the design of the AD step remained unaltered. Although the biogas production decreased by 4%, the electricity generated and the district heat produced increased by 14% and 12%, respectively, which resulted in the same overall energy efficiency as for scenario B and an ethanol production cost of 4.28 SEK/L. Although the ethanol production cost in the two sub-scenarios increased compared with scenario B, it did not exceed the ethanol production cost in the reference case (5.14 SEK/L).

### Sensitivity to co-product prices

**Figure 5:**
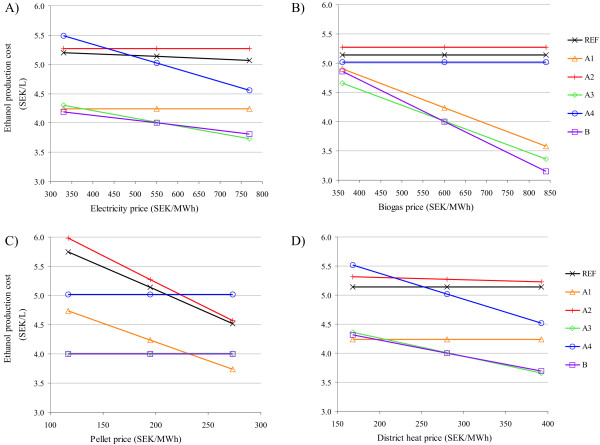
**Ethanol production cost (SEK/L) as a function of co-product prices**. Co-products: (A) electricity, (B) upgraded biogas, (C) pellets and (D) district heat. A summary of the scenarios is given in Table 1. REF: reference case; SEK: Swedish kronor.

The sensitivity of ethanol production cost to changes in the prices of the co-products was monitored by changing the price of one co-product at a time from -40% to +40%. The results are shown in Figure 5. The effect of changing price of the co-products depends on the share of the given co-product with regard to the total income: the higher the share the greater the effect. The electricity price had the highest impact on scenario A4, followed by scenarios A3 and B. However, scenarios A3 and B had the lowest ethanol production cost in almost the whole range investigated. Without selling electricity certificates (200 SEK/MWh) scenario A1 would be the most profitable if the spot price of electricity decreased below 260 SEK/MWh. Scenario B was that most affected by the price of biogas. Even with a 40% lower price of biogas the scenarios including biogas upgrading (A1, A3, B) resulted in lower ethanol production costs than the scenarios including biogas burning (reference case, A2, A4), although the difference was much smaller. The income from biogas could also decrease due to a lower production of biogas than that assumed. However, as long as the product of the amount of biogas produced and the price does not decrease by more than 40%, the scenarios with biogas upgrading are more favourable from an economic point of view. However, there is nothing to suggest that the raw biogas production assumed in scenarios A1 to A4 is overestimated. In scenario B, it has also been shown that assuming zero degradation of the polysaccharides in AD resulted in a slight decrease in biogas production.

The pellet price had the highest impact on scenario A2. For a pellet price higher than 235 SEK/MWh scenario A1 would result in a lower ethanol production cost than any of the other scenarios. Finally, the district heat price had a considerable influence on scenarios A4, A3 and B, in descending order. Scenarios A3 and B had the lowest ethanol production cost when the price of district heat was higher than 195 SEK/MWh, whereas below this value scenario A1 became the most economical. Hence, scenario A1 would have the lowest ethanol production cost, in the case where there was no income from district heating - for example, the plant location would not be appropriate for district heating.

## Conclusions

A techno-economic model of anaerobic digestion of the stillage stream has been developed based on the individual degradation of each component and was used to study different process configurations with various co-product combinations in a spruce-to-ethanol process. In concordance with the results obtained by Wingren *et al*. [21], the heat demand of the scenarios with alternative stillage treatment (with anaerobic digestion of the stillage) was significantly lower than that for the reference case, which included evaporation of the liquid fraction of the stillage. Due to the reduced process heat demand, the overall energy efficiency was improved in the alternative stillage treatment scenarios compared with the reference case. The highest energy efficiencies were obtained in the scenarios including district heating. Although the energy efficiencies of these scenarios varied slightly, their ethanol production cost differed significantly. Hence, it can be concluded that high energy efficiency does not necessarily result in improved economics. The implementation of district heating enables high energy efficiency by utilizing the heat available at low temperatures. However, it restricts the location of the plant as there must be a demand for the heat available.

The prices of co-products may vary considerably and, therefore, sensitivity analyses were performed in order to monitor the change in ethanol production cost. Two of the scenarios, which had the same co-product combination (upgraded biogas, electricity and district heat), but different feeds to the AD (only the liquid fraction of the stillage or the whole stillage stream), resulted in the lowest ethanol production cost over a wide range of co-product prices. These scenarios responded very similarly to changes in the co-product prices, resulting in almost identical ethanol production costs - with this combination of co-products the feed to AD does not influence the process economics significantly. If the price of pellets is increased to a certain level, or district heating can not be implemented, the scenario including the production of pellets and upgraded biogas becomes the most profitable. Hence, it can be concluded that alternative stillage treatment with biogas upgrading proved to be a favourable option in many respects.

There is still a need for the further development of the model regarding the wastewater treatment stage in both AD and the following aerobic step. It would be useful to implement the management of the inorganic compounds that originate from the raw material or which are added for pH adjustment. This is essential for the recirculation of the process water in order to avoid the accumulation of these compounds in the process.

## Abbreviations

AD: anaerobic digestion; CHP: combined heat and power; COD: chemical oxygen demand; DM: dry matter: FPU: filter paper unit; SEK: Swedish kronor; SSF: simultaneous saccharification and fermentation; WIS: water-insoluble solid.

## Competing interests

The authors declare that they have no competing interests.

## Authors' contributions

ZB carried out the process simulations and economic evaluation and wrote the paper. ZB and GZ designed the study and analysed the results. GZ and KR reviewed the manuscript. All authors read and approved the final manuscript.
